# Optimizing the Thermal Treatment of Mining-Waste-Amended Clays for Ceramic Aggregates in Pavement Applications

**DOI:** 10.3390/ma18133180

**Published:** 2025-07-04

**Authors:** Murilo Miguel Narciso, Lisley Madeira Coelho, Sergio Neves Monteiro, Antônio Carlos Rodrigues Guimarães

**Affiliations:** 1 Department of Fortification and Construction, Military Institute of Engineering-IME, Praça General Tibúrcio, 80, Urca, Rio de Janeiro 22290-270, Brazil; madeiralisley@gmail.com (L.M.C.);; 2 Department of Materials Science, Military Institute of Engineering-IME, Praça General Tibúrcio, 80, Urca, Rio de Janeiro 22290-270, Brazil; snevesmonteiro@gmail.com

**Keywords:** mineralogical transformation, calcined clay, mining waste, ceramic aggregates, *α*-Treton

## Abstract

Mining activities generate large volumes of tailings with significant environmental impact but also the potential for sustainable reuse in construction materials. This study evaluates the production of ceramic aggregates from mixtures of clay, sand, and iron ore waste subjected to thermal treatment at temperatures ranging from 600 to 1100 °C. The influence of calcination temperature on mineralogical transformations and mechanical integrity was investigated using X-ray diffraction (XRD) and the α-Treton parameter, derived from standardized impact resistance testing. The results indicate that the formation of metakaolinite between 700 and 900 °C enhances mechanical resistance, while higher temperatures (>900 °C) lead to structural degradation, followed by partial recovery due to mullite crystallization. The α-Treton curve exhibited clear correlation with the phase changes identified by XRD, demonstrating its applicability as a low-cost, sensitive proxy for optimizing thermal activation. A simplified methodology is proposed to optimize the thermal activation of such materials by correlating firing temperature with mineralogical evolution and mechanical integrity, contributing to the development of sustainable ceramic aggregates for pavement applications.

## 1. Introduction

The search for alternative construction materials has intensified worldwide, with the aim of reducing natural resource extraction and minimizing environmental impacts. In countries with a tropical climate, the availability of coarse aggregates is often limited due to either the absence of superficial geological deposits or the considerable distances between the sources of the material and the urban centers. This scenario creates the need to identify and develop sustainable solutions for the production of artificial aggregates [1].

Among the studied alternatives, thermally treated clay-based aggregates have been investigated as substitutes for natural aggregates, particularly in contexts where lightweight or structurally optimized materials are desired [2,3]. These aggregates, produced by firing specific clays at elevated temperatures, may exhibit reduced density, enhanced porosity, or improved bonding properties, depending on processing parameters [4]. Although expanded clays have been explored mainly for structural concretes and insulation [5], the calcination of clay mixtures for dense ceramic aggregates suitable for pavement applications has emerged as a promising path [1,6,7], especially when industrial or mining residues are incorporated [8,9]. In parallel, calcined clay has also been widely studied for its pozzolanic potential, as thermal activation induces mineralogical transformations that enhance reactivity [10,11,12].

According to ASTM C125 [13], a pozzolanic material is defined as one that, while having little or no cementitious value by itself, reacts with calcium hydroxide in the presence of moisture to form compounds with cementitious properties. This characteristic has encouraged its use as a supplementary cementitious material (SCM), contributing to the reduction in the clinker content in mixed cements and consequently CO_2_ emissions in the cement and concrete industry [14,15,16,17].

In addition to its use as a supplementary cementitious material, the calcination of clays, especially those with kaolinitic content, has been highlighted as a strategy for reducing environmental impacts in the construction industry. Thermal treatment between 600 °C and 850 °C promotes dehydroxylation and the formation of amorphous aluminosilicate phases with high pozzolanic activity, such as metakaolinite [18,19,20]. Although high-purity kaolinitic clays are limited to specific regions, low-grade or impure clays (with kaolinite content below 40%) are more widely available and economically viable, especially in developing countries [21]. Their utilization not only contributes to improved mechanical and durability performance in cementitious systems but also aligns with zero-waste mining principles by enabling the valorization of abundant mineral resources and industrial residues. Furthermore, replacing part of the clinker with calcined clay in mixed cements can significantly reduce CO_2_ emissions, given that cement production accounts for approximately 8% of the total global anthropogenic emissions [22,23,24]. Although the focus of this study is on the production of artificial aggregates, understanding thermal behavior, mineral transformations, and energy efficiency remains essential to optimizing the calcination parameters and ensuring material performance.

Beyond its application as a cement additive, calcined clay can be utilized as an artificial aggregate for paving, representing a viable alternative for tropical regions where natural aggregate availability is limited. In Brazil, the Army pioneered the production of such aggregates to address the scarcity of stone materials in the Northern region, where local geology hinders conventional aggregate acquisition [25,26]. In the Amazon region, where obtaining coarse aggregates is particularly challenging due to the predominance of residual soils and highly weathered rocks, the use of sintered calcined clay aggregates can reduce dependence on natural materials and long-distance aggregate transportation [9,27,28]. Additionally, by incorporating local clays and mining residues, this approach can lower production costs and minimize the environmental burden of waste accumulation, especially in regions lacking access to high-quality aggregates.

Within this context, Batista [29] investigated the use of artificial calcined clay (ACC) in Amazon region paving and concluded that mixtures containing clay calcined at 900 °C for 30 min exhibited a suitable resilience modulus, differing from values obtained for local soil. Silva et al. [9] corroborated these findings by demonstrating that asphalt mixtures with sintered ACC in asphalt concrete showed mechanical behavior comparable to natural aggregates while also reducing carbon footprint. Santos [8] analyzed the use of ACC as coarse aggregate and observed that asphalt mixtures with this material presented higher mechanical strength and lower permanent deformation compared to those using pebbles, common in the Amazon region. Furthermore, increasing the firing temperature improved component adhesion and reduced asphalt binder consumption due to the crystalline-to-amorphous phase transition [28,30], reinforcing ACC’s viability as a technically and environmentally sustainable paving alternative.

This approach, which promotes the use of calcined clay as an artificial aggregate, is consistent with zero-waste mining principles and supports the reuse of industrial byproducts in pavement applications. Recent studies have explored diverse sustainable alternatives in this context. For instance, Friber et al. [26] investigated the incorporation of calcined aggregates containing mining waste into multiple pavement layers, such as base, sub-base, and asphalt surfaces, confirming their technical viability. Similarly, Ma et al. [31] evaluated artificial aggregates derived from steel slag and fly ash processed by accelerated carbonation and reported improved microstructure and performance in asphalt mixtures, especially with respect to moisture and skid resistance.

Research has also emphasized the potential of these materials to reduce landfill disposal and promote circular economy strategies in construction [32,33,34]. In Brazil, multiple investigations have focused on the valorization of iron ore tailings in paving materials, indicating improved mechanical performance and environmental benefits [35,36,37]. Coelho et al. [38] further highlighted the feasibility of using steel slag aggregates optimized by the Bailey method in asphalt mixtures. Furthermore, Mondem and Balunaini [39] proposed the production of artificial aggregates from the overburden of the coal mine, reinforcing the applicability of the mining residues in different geotechnical contexts.

In the base layers of the pavement, calcined clay aggregates have also demonstrated favorable results. Barbosa et al. [25] showed that lateritic soil mixtures containing calcined aggregate 30% achieved adequate stabilization of the gradation of the structural layers. The production of these aggregates requires specific processing methods and raw material criteria. Cabral et al. [40] proposed a method for the production of calcined clay aggregate using clays with a plasticity index greater than 15% and granulometric compatibility with regions B, C, or D of the Winkler diagram, supported by formulations in studies such as Monteiro and Vieira [41], and Amaral et al. [42].

Beyond particle size and plasticity, pozzolanic reactivity plays a critical role in aggregate performance. According to Pinheiro et al. [12], standard tests such as the Frattini and modified Chapelle methods are widely used for this assessment, along with strength tests in cementitious matrices. Mineralogical techniques such as X-ray diffraction (XRD) and thermogravimetric analysis (TGA) remain essential tools to correlate mineral phase development with mechanical behavior.

Despite advances in calcined clay aggregate production and application, determining the optimal calcination temperature remains challenging as it influences not only pozzolanic activation and mechanical behavior but also new mineral phase formation, firing shrinkage, and process energy efficiency [33]. Furthermore, the incorporation of mining waste in ceramic and cementitious materials has been studied as a sustainable alternative, contributing to proper waste disposal, reduced environmental impacts, and the enhanced technical feasibility of artificial aggregates. Studies indicate that these wastes can be used in the production of ceramic bricks, demonstrating mechanical and environmental viability [43,44,45]. In addition, thermal processes can enhance their reactive properties. However, there remains a lack of research that correlates calcination temperature with mechanical behavior and material reactivity for pavement construction applications, underscoring the need for additional investigations focused on optimizing thermal activation conditions tailored to the mechanical requirements of pavement applications.

Facing these challenges, this study investigates the feasibility of producing artificial aggregates for pavement applications from calcined clay mixtures incorporating iron mining waste. This approach addresses two critical objectives: the sustainable reuse of mineral waste, often disposed of in large volumes and the development of cost-effective ceramic formulations for infrastructure use. The materials were proportioned using a matrix-based optimization aligned with the Winkler diagram to ensure granulometric compatibility. The study focuses on the influence of calcination temperature on the mechanical behavior of the aggregate, introducing the α-Treton parameter as a simplified tool to correlate thermal treatment with mechanical performance. The central hypothesis is that combining clay with mining residue and subjecting the mixture to optimized calcination can yield technically robust and environmentally responsible artificial aggregates for use in flexible pavement layers.

## 2. Materials and Methods

### 2.1. Materials

For the production of artificial calcined clay aggregates (ACCAs), three main components were used: sandy soil, iron ore tailings (red mud), and clay soil. The sandy soil was collected in Itacoara, Rio de Janeiro, Brazil; the iron ore waste originated from Itabirito, Minas Gerais, Brazil, a region with intensive mining activity; and the clayey soil was obtained from a municipal landfill in Dourados, Mato Grosso do Sul, Brazil.

These source materials, sandy soil (A1), iron ore waste (A2), and clayey soil (A3), were combined into a single optimized mixture for further processing and testing.

### 2.2. Methodology

**Figure 1:**
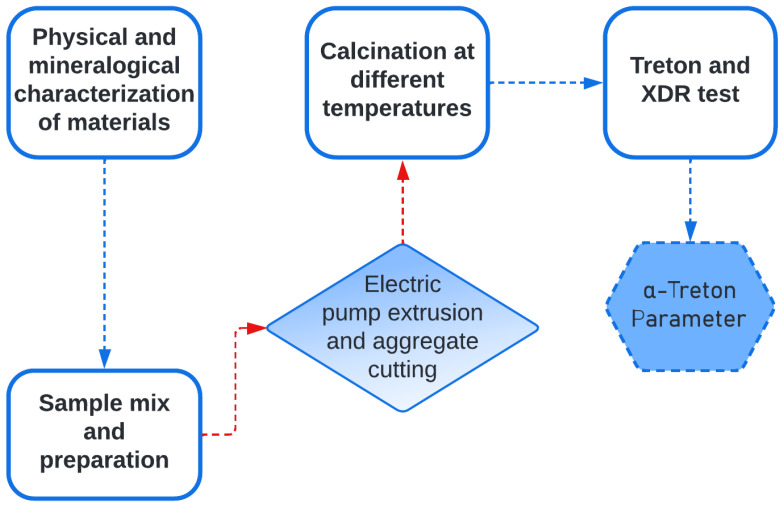
Methodology flowchart.

The methodological process of this study comprised the selection and characterization of raw materials, followed by the extrusion and calcination of the mixture, with the subsequent physical, mineralogical and mechanical evaluation of the aggregates produced. Figure 1 presents a flowchart that illustrates the main stages of the study.

**Figure 2:**
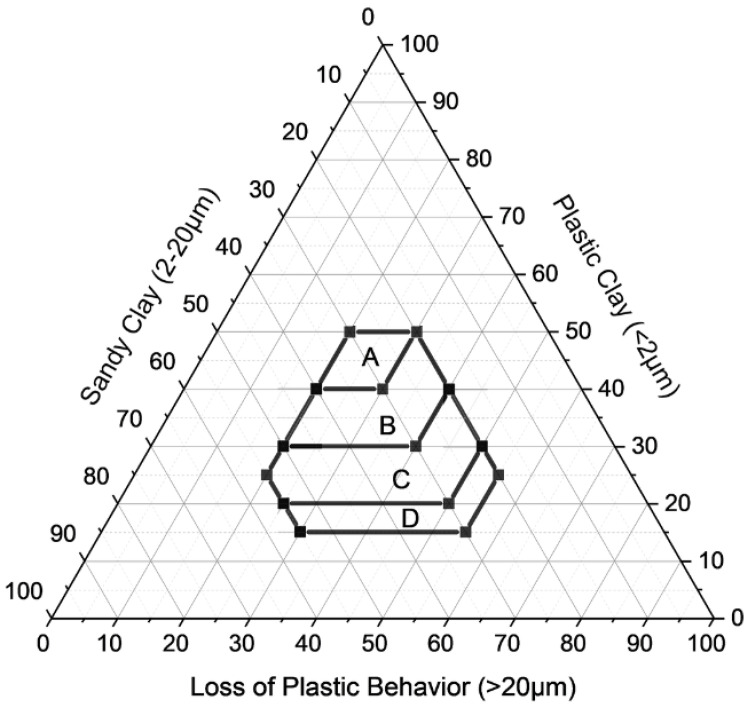
Winkler diagram triangle adapted from [47].

Based on previous studies by Cabral [46] conducted at the Military Institute of Engineering (IME), two criteria were established to define high-quality calcined clay, (i) a plasticity index (PI) greater than 15% and (ii) granulometric compliance with the Winkler diagram [47], to classify soils intended for calcination and ceramic production according to the appropriate grain sizes zones for red ceramic products, as shown in Table 1 and Figure 2.

To determine the optimal composition of the mixture, the particle size distributions of each material were analyzed and classified into three fractions: clay (<2 µm), silt (2–20 µm), and sand (>20 µm). The goal was to match the resulting blend to the centroid of Region A in the Winkler diagram (Table 1), which corresponds to high-quality ceramic formulations. The central values of Region A were adopted as target proportions:

$$\left\{\begin{array}{cc} \mathrm{Clay}\;\mathrm{fraction}: & 45\%\\ \mathrm{Silt}\;\mathrm{fraction}: & 30\%\\ \mathrm{Sand}\;\mathrm{fraction}: & 25\% \end{array}\right.$$

Let *x*, *y*, and *z* represent the mass fractions (in decimal form) of samples A1, A2, and A3, respectively. The particle size distribution of each raw material was experimentally determined and organized in the following structure:
MaterialClay (%)Silt (%)Sand (%)*ine*Sandy soil (A1)*a*_11_*a*_12_*a*_13_Waste (A2)*a*_21_*a*_22_*a*_23_Clayey soil (A3)*a*_31_*a*_32_*a*_33_


A system of linear equations was then solved to determine the values of *x*, *y*, and *z* that best fit the target profile:

$$\left\{\begin{array}{c} a_{11}x+a_{21}y+a_{31}z=45\\ a_{12}x+a_{22}y+a_{32}z=30\\ a_{13}x+a_{23}y+a_{33}z=25\\ x+y+z=100 \end{array}\right.$$

Solving this system yielded the following proportions:

x=14%,y=12%,z=74%

This optimized blend ensured granulometric compatibility with the Winkler diagram and practical workability for forming and calcination, justifying its selection as the baseline mixture for the experimental campaign.

### 2.3. Physical Characterization

The soils and waste were characterized through grain size analysis by sieving [48] and sedimentation [49] as well as specific gravity [50], moisture content [51], and consistency limits [48,52].

### 2.4. Mineralogical Characterization

The mineralogical composition of the samples was analyzed by X-ray diffraction (XRD) using a Malvern Panalytical diffractometer with CoKα anode, operating at 40 mA and 40 kV, with scanning between 10° and 90°. The diffraction patterns were interpreted by comparison with standard crystallographic cards from the ICDD database.

### 2.5. Sample Preparation and Calcination

**Figure 3:**
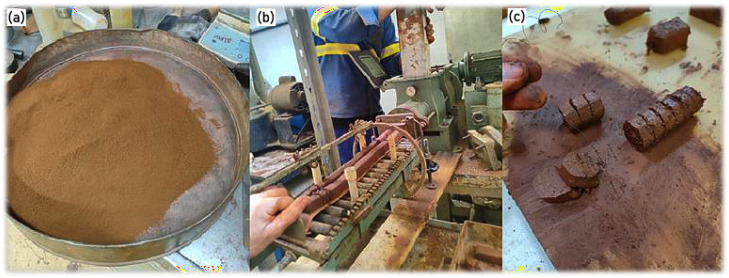
Manufacturing stages: (**a**) raw material mixing; (**b**) electric compactor extrusion; (**c**) nylon wire cutting.

The samples were prepared using an electric soil compactor available at the IME Soil Laboratory. Initially, the raw materials were mixed and extruded using the compactor. The extruded material was then cut with nylon wire into square-shaped samples measuring 15 mm × 15 mm × 10 mm (length × width × thickness). Following this, the samples were air-dried at room temperature for seven days to reduce moisture content and ensure better thermal behavior during subsequent calcination. Figure 3 illustrates the main steps involved in this process.

After drying, the samples were subjected to a calcination process at temperatures ranging from 600 to 1100 °C, with a controlled heating rate of 10 °C/min. The temperature of 600 °C was chosen as the reference point as it marks the onset of kaolinite dehydroxylation [53], a critical transformation that increases the material’s pozzolanic reactivity. This mineralogical change improves the mechanical performance of the material, as highlighted by Pinheiro et al. [12].

### 2.6. α-Treton Parameter

To assess the influence of calcination temperature on the mechanical behavior of the treated clay aggregate, this study introduces the α-Treton parameter, an experimental indicator derived from the impact resistance test described in the DNER-ME 399 standard [54]. This parameter quantifies the material’s loss under impact stress and serves as a proxy for its structural integrity and pozzolanic activation potential.

According to the standard procedure, the Treton impact test evaluates the mechanical integrity of aggregate specimens by subjecting them to a series of controlled vertical impacts using a standardized apparatus. After testing, the material is sieved through a 1.7 mm mesh, and the percentage of mass lost interpreted as impact-induced fragmentation is calculated using Equation (1):

(1) $$T=\left(1-\frac{M_{r}}{M_{1}}\right)\times 100$$

where

*T* = impact loss (%), also referred to as the α-Treton value;
$M_{1}$ = initial mass of the sample (g);
$M_{r}$ = mass of material retained on the 1.7 mm sieve after impact (g).

The final α-Treton value is defined as the average of three independent measurements for each calcination temperature. In this study, 45 aggregate units were tested per temperature, following the standard. Samples were thermally treated at temperatures ranging from 600 °C to 1100 °C, in 50 °C increments, and then subjected to the Treton test to evaluate their mechanical behavior post calcination.

To explore the mechanical implications of the mineralogical transformations induced by calcination, the α-Treton parameter was adopted as a practical indicator. This parameter reflects the residual mechanical integrity of the thermally treated aggregates and was analyzed alongside XRD data. By comparing the trends in α-Treton values across different calcination temperatures with the corresponding phase transitions—such as the dehydroxylation of kaolinite, formation of metakaolinite, and subsequent crystallization of mullite, it becomes possible to assess the mechanical consequences of each transformation. This integrated approach aims to provide a simplified yet meaningful understanding of how thermal treatment affects both the structural and functional behavior of calcined clays, facilitating the selection of optimal activation conditions for use in pavement materials.

Although XRD analyses were performed for all calcination temperatures (600–1100 °C, in 50 °C increments), only selected patterns at 700, 850, and 1100 °C are presented as they most clearly represent the main mineralogical transitions. This approach minimizes redundancy while highlighting key structural transformations, which are subsequently correlated with the evolution of the α-Treton parameter and the theoretical model of kaolinite transformation. Together, these elements provide a coherent basis for interpreting the effects of thermal treatment on both mineralogical phases and mechanical behavior.

## 3. Results and Discussion

### 3.1. Physical Characterization

**Figure 4:**
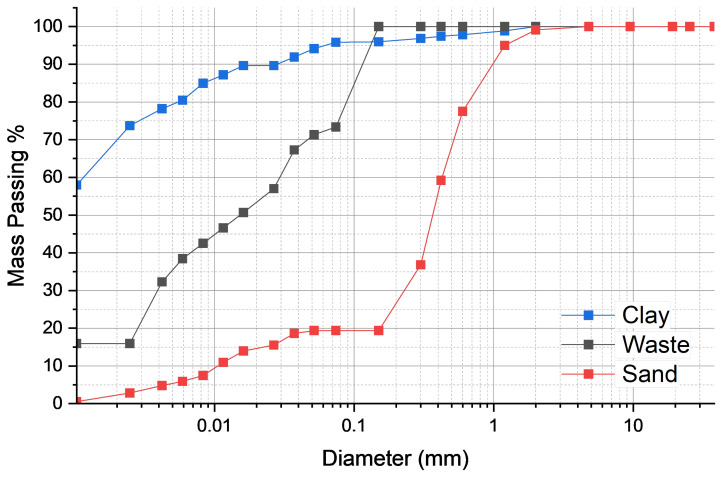
Particle size distribution curves of the samples.

The physical characterization of the raw materials is presented in Table 2. Granulometric analysis of the materials was performed through sieving and sedimentation, and the resulting particle size distribution curves are shown in Figure 4. These curves allow a visual comparison of the particle gradation across the three components: A1 (sandy), A2 (waste), and A3 (clay).

In addition to the graphical analysis, Table 3 presents the statistical descriptors derived from the cumulative curves, including d_10_, d_50_, d_90_, and the mean diameter (D_mean_). These values quantify the fineness and distribution characteristics of each material.

The values confirm the role of each constituent in the mixture. The clay has an extremely fine gradation (d_50_ = 0.002 mm), while the waste exhibits intermediate particle size, consistent with silt fractions (d_50_ = 0.0085 mm), and the sand shows coarser characteristics (d_50_ = 0.16 mm). This composition reflects an engineered blend suitable for ceramic formulation.

### 3.2. Winkler Classification

**Figure 5:**
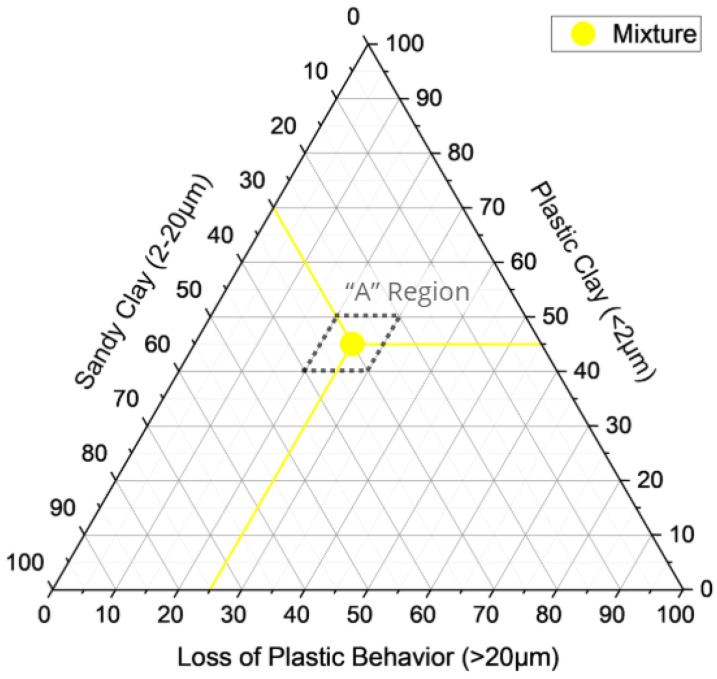
Winkler diagram of the mixture.

The optimized blend was positioned on the Winkler ternary diagram, which categorizes ceramic mixtures based on the proportion of plastic clay (<2 µm), sandy clay (2–20 µm), and particles that result in loss of plastic behavior (>20 µm). As shown in Figure 5, the mixture falls within Region A, which corresponds to formulations with ideal granulometry for producing high-quality ceramic products.

This classification is consistent with the particle size statistics reported previously, reinforcing the technical suitability of the material blend. The mixture balances the three granulometric fractions effectively, achieving a structure favorable to compaction, formability, and calcination.

The plasticity index (PI) of the final blend was not directly measured but estimated based on the PI values of the individual soils, resulting in an approximate value of 13%. According to Cabral [46], ceramic formulations for calcination should exhibit a PI greater than 15% to ensure adequate workability. Despite that, Vieira et al. [55] consider PI values ≥10% to be acceptable for ceramic shaping processes. Therefore, considering the favorable granulometry and alignment with Winkler’s Region A, the mixture remains viable for ceramic production, although some limitations in moldability may arise depending on the shaping technique employed. In this regard, Friber et al. [26] reported extrusion failures when using mining waste with low PI, confirming the need to limit its proportion in mixtures to ensure proper forming. Similarly, Polivanov et al. [6] emphasized that insufficient plasticity can hinder shaping by increasing cracking susceptibility and reducing particle packing, which may negatively impact sintering behavior and final densification. Hence, while technically acceptable, the PI discrepancy may affect processing efficiency and should be carefully considered in practical application.

### 3.3. Mineralogical Analysis

In the clayey soil sample (A3), the main crystalline phases identified by XRD were kaolinite, quartz, and hematite. The presence of kaolinite was qualitatively confirmed by characteristic peaks, notably around 14.8° and 25.0° 2θ, associated with its layered silicate structure. The absence of the basal reflection at 12.3° 2θ (kaolinite 001) may indicate structural disorder or the presence of interstratified kaolin-group minerals, such as halloysite. While no quantitative phase analysis was performed, the XRD pattern exhibited clear signals of these clay minerals, suggesting that they may influence the thermal behavior of the mixture. The iron ore waste (A2) showed a predominance of quartz and iron oxides, particularly hematite, as evidenced by intense peaks near 33°, 35°, 49.5°, and 63° 2θ. Quartz peaks were also clearly observed. Although not explicitly labeled in the diffractogram, minor peaks suggest the possible presence of accessory minerals such as goethite, manganese oxides, and calcium carbonates. Signals in the region of 60.7° 2θ may be attributed to goethite, while peaks around 44.6° and 35.2° 2θ may be related to manganese phases and carbonates, respectively. Additional signals potentially associated with phosphorus- and aluminum-bearing phases were also observed. These may result from residual apatite or gibbsite, likely originating from weathering or hydrothermal alteration processes. Although present in minor quantities, these accessory minerals can affect the reactivity and environmental behavior of the tailings, particularly under leaching conditions [56,57,58]. The sandy soil (A1) exhibited a predominantly quartz composition, as indicated by sharp and intense peaks, especially near 26.6° 2θ. Secondary peaks around 38.2° and 12.2° 2θ suggest the possible presence of feldspar and mica, respectively, which are typical constituents of silicate-rich sandy soils.

**Figure 6:**
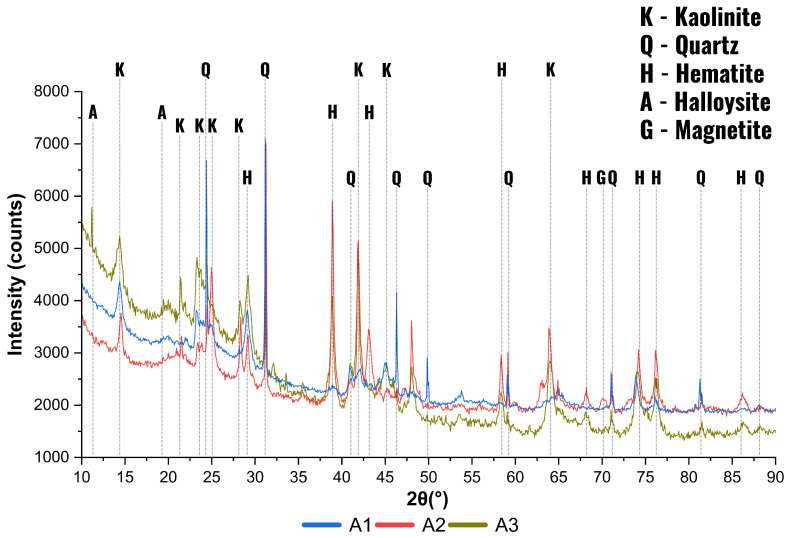
XRD patterns of the raw materials: A1 (sandy soil), A2 (iron ore waste), and A3 (clayey soil). Identified phases include kaolinite (K), quartz (Q), and hematite (H).

Following the analysis presented in Figure 6, to assess the influence of thermal treatment, XRD analyses were also conducted on calcined clayey soil samples. Representative diffraction patterns obtained at selected temperatures are discussed in Section 3.4, along with the interpretation of the α-Treton parameter, which integrates the mineralogical and mechanical behavior.

### 3.4. α-Treton Parameter

**Figure 7:**
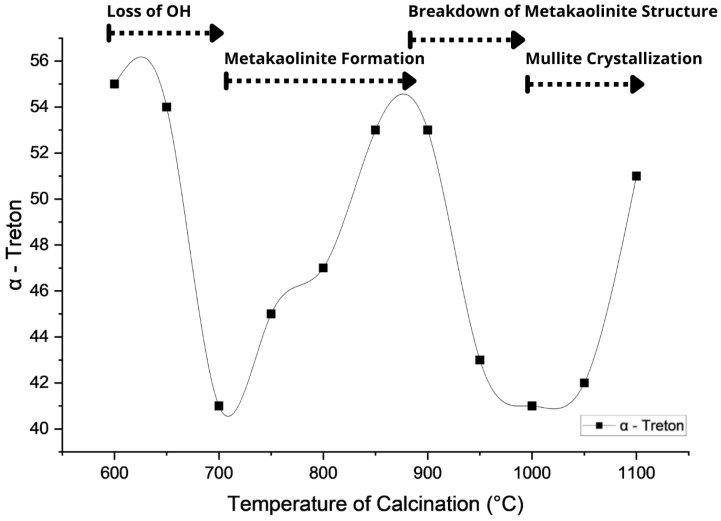
α-Treton curve correlating mechanical and mineralogical behavior with calcination temperature.

The influence of calcination temperature on the mechanical integrity of the thermally treated clay mixture was evaluated using the α-Treton parameter, derived from standardized impact resistance testing. As shown in Figure 7, this parameter effectively captures the structural variation of the aggregates with temperature and exhibits a strong correspondence with the typical mineralogical transformations of kaolinitic systems. Consequently, it serves as a simplified yet informative indicator of pozzolanic activation and mechanical performance.

The results indicate that the α-Treton values ranged from approximately 40% to 56%, representing the proportion of mass lost due to impact. Lower α-Treton values reflect a greater retention of mass after impact and, therefore, higher mechanical resistance. Depending on the calcination temperature, between 44% and 60% of the initial aggregate mass was retained, indicating variable but generally moderate to good structural integrity throughout the thermal treatment range. The α-Treton curve reveals distinct patterns that align with key mineralogical transformations, allowing its behavior to be segmented into four thermal intervals:

(i) **600–700 °C:** A reduction in α-Treton values is observed, attributed to the initial dehydroxylation of kaolinite, which weakens the lamellar structure and increases porosity. Recent findings by Fernandez and Snellings [59] demonstrate that this structural disruption temporarily reduces mechanical cohesion.
(ii) **700–900 °C:** A progressive increase in mechanical performance is noted, linked to the formation of metakaolinite, a highly reactive amorphous aluminosilicate. According to Rasmussen et al. [60], this phase contributes to particle agglomeration and matrix densification in ceramic systems.
(iii) **900–1000 °C:** A decline in α-Treton values occurs, associated with the collapse of metakaolinite and the emergence of spinel-type phases and amorphous silica. This behavior is consistent with the results of Duxson et al. [61], who relate this transformation to internal structural disorder and the porosity increase.
(iv) **Above 1000 °C:** A slight recovery in the parameter is detected, attributed to the crystallization of mullite and cristobalite. These phases contribute to structural integrity and long-term mechanical strength, as reported by Pereira et al. [62].

To complement the discussion and provide a clearer understanding of how mineralogical transformations influence mechanical behavior, Table 4 summarizes key thermal intervals, associated phases, structural characteristics, and their implications for aggregate resistance. This comparative synthesis helps distinguish the calcination range required for pozzolanic activation from that typically employed in high-temperature sintering for other ceramic purposes, thereby reinforcing the applicability of moderate temperature treatment in sustainable aggregate production strategies.

Additionally, based on the observed results, it is proposed that mixtures exhibiting α-Treton values equal to or below approximately 45%, which corresponds to at least 55% of the mass being retained after impact, may be preliminarily considered mechanically viable for use as artificial aggregates. This empirical threshold reflects increased structural integrity and resistance to fragmentation, and may serve as a practical guideline to support thermal treatment decisions in systems with similar mineralogical compositions.

**Figure 8:**
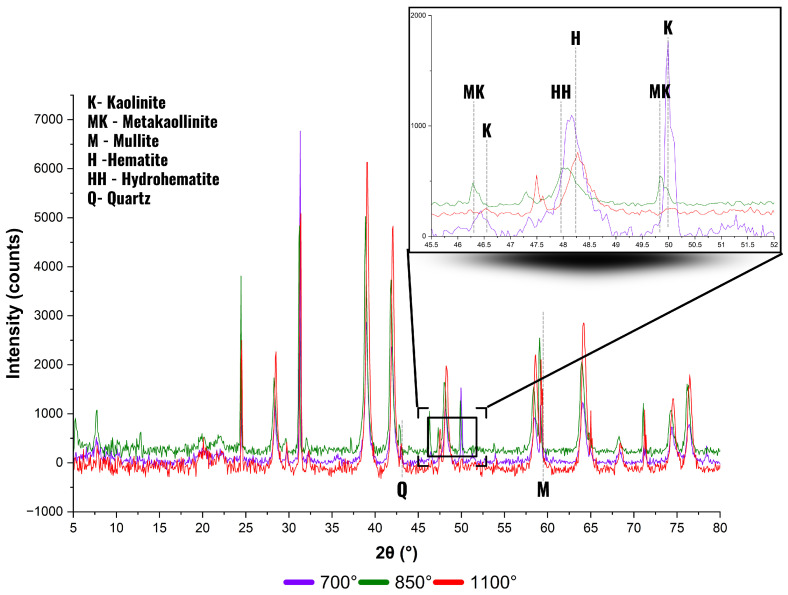
XRD patterns of samples calcined at 700 °C, 850 °C, and 1100 °C, indicating progressive transformation from kaolinite to amorphous and crystalline high-temperature phases.

To support the mechanical findings, Figure 8 presents the XRD patterns of the calcined samples at 700 °C, 850 °C, and 1100 °C, temperatures that represent key stages in the mineralogical evolution of the clayey mixture. To enhance the interpretation of phase transitions, a magnified region between 45° and 52° 2θ is included. This interval was selected because it concentrates key diffraction signals associated with the transformation of kaolinite into high-temperature phases. Specifically, it captures the progressive disappearance of kaolinite, the emergence of metakaolinite traces, and the crystallization of hematite and hydrohematite, transitional phases relevant to thermally treated iron-bearing clays. Due to the partial overlap and low intensity of some of these peaks, the zoomed view facilitates a clearer identification of these mineralogical changes that are not easily distinguishable in the full-scale diffractogram.

At 700 °C, kaolinite peaks remain visible, particularly near 46.5° and 49.8° 2θ, indicating incomplete dehydroxylation at this stage. This persistence has been reported in other kaolinitic systems, especially when the clay minerals are embedded in iron-rich matrices or subjected to slow heating, delaying full conversion into metakaolinite [33,63,64,65]. In this stage, a broad feature near 35–36° 2θ is attributed to hydrohematite, a transitional phase formed during the partial dehydration of goethite or ferrihydrite. This observation aligns with reports of mineralogical transformations in thermally treated iron-bearing clays, where hydrohematite often precedes complete hematite crystallization [33,56].

At 850 °C, the disappearance of kaolinite peaks and the development of a diffuse halo confirm the predominance of an amorphous phase, identified as metakaolinite. However, subtle peaks in the range of 46°–50° 2θ suggest that some ordering or residual structure may still persist.

At 1100 °C, sharp peaks emerge, especially around 58°, 66°, and 75° 2θ, indicating the formation of mullite and well-crystallized hematite. These transformations are consistent with the expected evolution of thermally activated clays and help explain the changes observed in the mechanical behavior of the samples, particularly as reflected in the α-Treton curve.

**Figure 9:**
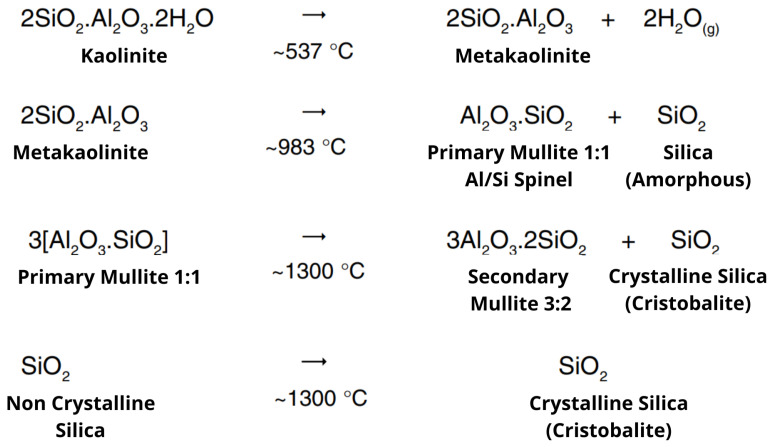
Thermal transformations of kaolinite and associated phase changes during calcination. Adapted from classical mineralogical reaction models [64].

Although XRD analyses were conducted at 50 °C intervals from 600 °C to 1100 °C, only the patterns at 700 °C, 850 °C, and 1100 °C are shown in Figure 8 as they best represent the main transformation stages and reduce visual redundancy. The interpretation of these transformations is further supported by the theoretical model presented in Figure 9, which illustrates the sequential mineralogical evolution of kaolinite under thermal treatment, following the classical reaction framework proposed by Souza Santos [64].

The convergence between the mechanical behavior and the mineralogical transformations observed across the calcination temperature range substantiates the robustness of the α-Treton parameter as a rapid and cost efficient proxy for evaluating the degree of pozzolanic activation in thermally treated kaolinitic systems. Despite its application being restricted to a specific kaolinitic clay composition modified with mining waste, the reproducibility and consistency of the results underscore the potential of the α-Treton parameter as a practical decision-making tool for optimizing calcination protocols in the engineering of ceramic aggregates intended for pavement layers. To enhance the generalizability and technical robustness of this methodology, further investigations encompassing a broader spectrum of clay mineralogies and physicochemical profiles are recommended.

#### Application Strategy and Optimization Guidelines

**Figure 10:**
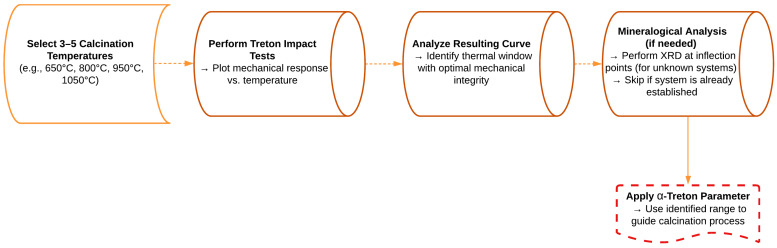
Recommended workflow for using the α-Treton parameter in optimizing calcination for ceramic aggregates.

To facilitate the practical application of the α-Treton parameter in various clay residue systems, a simplified experimental workflow is proposed (Figure 10). The method involves selecting three to five representative calcination temperatures across the thermal spectrum (e.g., 650 °C, 800 °C, 950 °C, 1050 °C), followed by Treton impact testing.

The resulting curve enables the identification of the thermal window that yields optimal mechanical integrity for ceramic aggregate production. For systems with unknown mineralogical profiles, targeted XRD analyses at the main inflection points are recommended to confirm the correlation between mechanical performance and structural transformations. In established systems, however, the α-Treton parameter alone may be sufficient to guide the calcination process.

## 4. Conclusions

This study demonstrated the technical feasibility of producing artificial ceramic aggregates from a clay mining waste mixture through controlled thermal treatment. The mechanical and mineralogical characterization confirmed that calcination temperature has a decisive influence on both phase transformations and structural integrity of the material.

The main conclusions are as follows:

- The proposed α-Treton parameter proved to be a practical, sensitive, and low-cost proxy to evaluate the mechanical integrity of calcined aggregates. Its behavior aligned closely with the mineralogical changes typically observed in kaolinitic systems.
- The temperature range between 700 and 900 °C was identified as optimal for the formation of metakaolinite, corresponding to improved impact resistance. Outside this range, particularly above 900 °C, structural degradation and recrystallization processes reduce aggregate integrity.
- Although XRD analysis supported the interpretation of mineral phase evolution, the α-Treton parameter alone may be sufficient for routine optimization of calcination in systems with known mineralogy. For heterogeneous or unexplored compositions, complementary mineralogical validation is recommended at key thermal inflection points.
- A simplified experimental workflow based on the α-Treton curve was proposed to streamline the process design across different raw material systems, providing a replicable framework for thermal optimization in ceramic aggregate production.

In summary, this research presents a reproducible methodology for optimizing the thermal activation of clay residue mixtures for sustainable aggregate production, reinforcing their potential for use in pavement layers. Future studies should explore the application of advanced mechanical testing methods, such as repeated load triaxial (RLT) tests, to assess the mechanical behavior of thermally treated aggregates under realistic induced stress conditions and complement the α-Treton evaluation and further substantiate the mechanical behavior of thermally treated aggregates. Additionally, further investigations with different mixture compositions and reference materials (e.g., pure clay) are encouraged to expand the generalizability and robustness of the proposed methodology.

## Figures and Tables

**Table 1:** Grain size distribution of red ceramic products according to the Winkler diagram [47].

Region	Clay (<2 μm)	Silt (2–20 μm)	Sand (>20 μm)
A—Quality ceramics	40–50%	20–40%	20–30%
B—Roof tiles and covers	30–40%	20–50%	20–40%
C—Perforated bricks	20–30%	20–55%	20–50%
D—Solid bricks	15–20%	20–55%	20–55%

**Table 2:** Characteristics of materials used.

Test	A1	A2	A3
Specific gravity	2.69	3.36	2.77
Liquid limit (%)	43.4	43.9	44.2
Plastic limit (%)	34.1	31.0	31.3
Plasticity index (%)	9.3	12.9	12.9

**Table 3:** Statistical descriptors of particle size distribution.

Material	d_10_ (mm)	d_50_ (mm)	d_90_ (mm)	D_mean_ (mm)
A1	0.0020	0.0020	0.0500	0.0162
A2	0.0020	0.0085	0.2000	0.0518
A3	0.0500	0.1600	0.5000	0.2376

**Table 4:** Relationship between calcination temperature, mineralogical transformations, structural nature, and mechanical resistance of the aggregate.

Temperature Range (°C)	Main Phase Transformation	Structural Nature	Impact on Mechanical Resistance
600–700	Initial kaolinite dehydroxylation	Partially disordered	Decrease—temporary weakening due to structural breakdown
700–900	Formation of metakaolinite	Amorphous and cohesive	Increase—enhanced impact resistance due to structural reorganization
900–1000	Metakaolinite breakdown; spinel and amorphous silica	Unstable and disrupted	Decrease—reduced integrity due to phase disintegration
>1000	Onset of mullite crystallization	Crystalline and dense	Moderate recovery—formation of mechanically robust phases

## Data Availability

The original contributions presented in this study are included in the article. Further inquiries can be directed to the corresponding author.
